# Novel pathogenic variants in *CUBN* uncouple proteinuria from renal function

**DOI:** 10.1186/s12967-022-03706-y

**Published:** 2022-10-20

**Authors:** Chun Gan, Xindi Zhou, Dan Chen, Huan Chi, Jiawen Qiu, Hui You, Yaxi Chen, Mo Wang, Haiping Yang, Wei Jiang, Qiu Li

**Affiliations:** 1 Pediatric Research Institute, Department of Nephrology, Ministry of Education Key Laboratory of Child Development and Disorders, National Clinical Research Center for Child Health and Disorders, China International Science and Technology Cooperation Base of Child Development and Critical Disorders, Chongqing Key Laboratory of Pediatrics, Children’s Hospital of Chongqing Medical University, Chongqing, People’s Republic of China; 2 Centre for Lipid Research & Key Laboratory of Molecular Biology for Infectious Diseases (Ministry of Education), Institute for Viral Hepatitis, Department of Infectious Diseases, the Second Affiliated Hospital, Chongqing Medical University, Chongqing, People’s Republic of China

**Keywords:** Pathogenic variants, *CUBN*, Amnionless, Proteinuria

## Abstract

**Background:**

Proteinuria is an unfavorable clinical condition highly associated with a risk of renal and cardiovascular disease in chronic kidney disease (CKD). However, whether all proteinuria forms are linked to renal impairment are still unclear. Cubilin is an endocytic receptor highly expressed in renal proximal tubules mediating uptake of albumin, transferrin and α1-microglobulin.

**Methods:**

Exome sequencing method initially identified candidate genes. With the application of exome sequencing combined with Sanger sequencing, we further focused on *CUBN* through bioinformatics analysis. The pathogenic effects of the potentially causative variants were verified utilizing complementary analysis of clinical data and systematic characterization of the variants’ expression and function with clinical samples and in vitro experiments in HEK293T cell lines along with in vivo experiments in mice.

**Results:**

In this study, we identified four novel variants locating after the vitamin B12 (vitB12)-binding domain of Cubilin (encoded by *CUBN*, NM_001081.3: c.4397G > A (p.C1466Y), c.6796C > T (p.R2266X), c.6821 + 3A > G and c.5153_5154delCT (p.S1718X)) in two families. Moreover, the variants severely affected the expression and function of Cubilin in renal proximal tubules and caused albuminuria, increasing levels in urine transferrin and α1-microglobulin, but without progressive glomerular filtration barrier (GFB) impairment, vitB12 deficiencies or abnormal blood levels of HDL and albumin. Further mechanistic insights showed that the variants after the vitB12-binding domain of *CUBN* merely disrupted the association with Amnionless (AMN) that exhibited aberrant localization in cell cytoplasm rather than membrane.

**Conclusions:**

Here, our findings suggested that different mutation types after the vitB12-binding domain of *CUBN* uncouple proteinuria from glomerular filtration barrier, that may be an unexpectedly common benign condition in humans and may not require any proteinuria-lowering treatment or renal biopsy.

**Supplementary Information:**

The online version contains supplementary material available at 10.1186/s12967-022-03706-y.

## Background

Chronic kidney disease (CKD) is a general clinical term encompassing a heterogeneous group of disorders affecting more than 10% of the world’s population. Patients with CKD are at risk of end-stage renal disease (ESRD) incorporating, diabetic kidney disease, hypertensive nephropathy, glomerulonephritis, and nephrotic syndrome [1–4]. As part of the etiology of these conditions, marked proteinuria is the core clinical manifestation. Proteinuria consists of elevated albumin, α1-microglobulin, β2-microglobulin levels in urine. Its role as an independent early risk factor for renal impairment is well established and mainly arises from disorders of the glomerular filtration barrier (GFB). The glomerular basement membrane (GBM), together with podocytes and endothelial cells, comprise this barrier between plasma proteins and urine [1]. However, albumin is not completely retained by the barrier. To prevent proteinuria, renal tubules are well adapted to reclaim albumin [5, 6]. Unfortunately, pathogenetic mechanisms involving genetic polymorphisms underlying renal tubule dysfunction and associated with chronic proteinuria remain largely unknown.

Fortunately, exome sequencing (ES), a first-line diagnostic method in several clinical disciplines, has become increasingly relevant for the identification of genetic factors in CKD progression [7]. Approximately 25% of patients with CKD report a family history, whereas Mendelian causes account for approximately 10% of adult ESRD cases, and are leading causes of nephropathy in children [8]. Moreover, renal tubules overloaded with protein caused by gene variants may cause dysfunction in tubular epithelial cells [9]. Albumin is reabsorbed along proximal tubules by receptor-mediated endocytosis including the candidate binding proteins, Cubilin and Megalin, that are present in endosomal/lysosomal protein degradative pathway [10]. Therefore, the identification of gene variants related to CKD development will improve our understanding of renal disease mechanisms.

Cubilin (*CUBN*) is a 460-kDa peripheral membrane glycoprotein and a multi-ligand endocytic receptor with significant physiological functions. Typically, the uptake receptor complex, consisting of Cubilin, Megalin, and Amnionless (AMN) is critical for the receptor-mediated tubular reabsorption of key ligands, such as albumin, transferrin and α1-microglobulin, β2-microglobulin, from glomerular ultrafiltrates, and intestinal uptake of vitB12 [10, 11]. Cubilin lacks a transmembrane domain and an intracellular domain, and thus requires AMN to function as a receptor complex, the absence of which will lead Cubilin to retained in the endoplasmic reticulum and unable to be targeted to the plasma membrane [12].

Cubilin deficiency fails to maintain blood levels of high-density lipoprotein (HDL) and albumin [13]. And mutations affecting either of the 2 proteins may abrogate function of the receptor complex and cause Imerslund-Gräsbeck syndrome (IGS) characterized by intestinal malabsorption of vitB12 and in some cases proteinuria [14]. In recent years, the identification of receptor dysfunction roles, mediated by genetic variants, has suggested not all proteinuria forms are damaging, and has provided key information for clinical diagnostics [15–17]. However, whether Cubilin deficiency caused by variants are all associated with IGS, abnormal blood levels of HDL or albumin still leaves some issues to be studied.

In this study, we described two probands had unexplained albuminuria, increased in urine transferrin and α1-microglobulin levels. Wonderingly, both probands failed to respond to proteinuria-lowering therapy. Using homozygosity mapping and ES, novel variants [NM_001081.3: c.4397G > A (p.C1466Y), c.6796C > T (p.R2266X)], c.5153_5154delCT (p.S1718X) and c.6821 + 3A > G) locating after the vitB12-binding domain of Cubilin were identified in two families. The variants were located near C-terminal CUB domains of Cubilin and led to Cubilin deficiency in expression and function. We have linked pathogenic *CUBN* variants near C-terminal CUB domains of Cubilin and chronic isolated proteinuria to autosomal recessive inheritance. Also, AMN acting as a chaperone for Cubilin to facilitate membrane localization, has aberrant localization in cytoplasm occurring with the variants. Thus, variant mechanisms considerably affected Cubilin expression and function in the probands accompanied by aberrant cytoplasmic localization of AMN but normal expression and localization of Megalin.

Importantly, the deficiency of Cubilin only caused albuminuria, increased in urine transferrin and α1-microglobulin levels, but without progressive podocyte injury as evidenced by normal podocyte-specific proteins (Synaptopodin and WT1), exhibiting normal blood levels of HDL and albumin without vitB12 malabsorption. The identification of variants and associated chronic isolated proteinuria may suggest variants locating after the vitB12-binding domain of Cubilin were not associated with the function of GFB, blood levels of HDL and albumin and vitB12 absorption. Also, our data may impact genetic counseling and functional validation for inherited CKD and associated conditions that may be an unexpectedly common benign condition in humans and may not require any proteinuria-lowering treatment or renal biopsy.

## Methods and materials

### Exome sequencing

Genomic DNA was obtained from whole blood using the QIAamp DNA Mini Kit (180134, Qiagen). 97 genes related to kidney disease were kept as a gene capture strategy, using the GenCap custom enrichment kit (MyGenostics Inc) following the manufacturer’s protocol. The enriched libraries were sequenced using an Illumina HiSeq 2000 sequencer (Illumina), which running for paired-end reads of 150 bp. The clean reads were aligned to the reference human genome (hg19) with Short Oligonucleotide Analysis Package (SOAP) aligner software (SOAP2.21; soap.genomics.org.cn/soapsnp. html). Afterwards, single nucleotide polymorphisms (SNPs) were annotated with the SOAPsnp program, and the deletions and insertions (InDels) were detected using Genome Analysis Toolkit software 3.7. Low-quality variations were filtered out using a quality score ≥ 20 and MAF ≤ 0.01 and the schematic of screening workflow were shown in Additional file 1: Fig. S1. All variants were verified by Sanger sequencing.

### Minigene assay

Vector pSPL3, known as the exon trapping vector, was carried out for minigene assay. Briefly, exon 44 of CUBN and its adjacent intron 43 and 44, was PCR-amplified using the following primers: forward 5′-accagaattctggagctcgagATTCATCTAT CAGAAACATGATATATT-3′ and reverse 5′-accagaattctggagctcgagCAATGAGAATAGATAAATGGTCTGGCA-3′. XhoI and NheI were chosen as restriction sites. The PCR products were inserted into the vector pSPL3 following the standard process with ClonExpress II one step cloning kit (C112, Vazyme). The mutant type was constructed according to the procedure by Mut Express II Fast Mutagenesis Kit V2 (C214, Vazyme). Wild-type and mutant types were transfected into HEK293T cells with lipofectamine 3000 (Life Technologies). RNA was harvested using the Steadypure Quick RNA Extraction Kit (AG21023, Accurate biology) at 24 h after transfection. Then cDNA synthesis was performed with HiScript III 1st strand cDNA Synthesis Kit (R312, Vazyme). Subsequently, cDNA was PCR-amplified using the following pSPL3 specific primers:SD6-5′-TCTGAGTCACCTGGACAACC-3′ and SA2-5′-ATCTCAGTGGTATTTGTGAGC- 3′. The PCR fragments were identified by Sanger sequencing to evaluate the alternative splicing.

### Bioinformatics analysis

Phylogenetic analysis of *CUBN* was used an online tool, the interactive tree of life (https://itol.embl.de). Schematic of the Cubilin protein domains was analyzed by the SMART (a Simple Modular Architecture Research Tool). ClustalW multiple sequence alignment of Cubilin protein in several species was achieved by Clustal Omega (https://www.ebi.ac.uk/Tools/msa/clustalo/). Crystal structures of CUB domain were analyzed by the SWISS-MODEL (https://swissmodel.expasy.org).

### Histological analysis and staining

The *cubn* patient together with the hospitalized patients of minimal change nephropathy (MCD) and focal segmental glomerulosclerosis (FSGS) identified as non-hereditary nephropathy with similar age to the proband underwent kidney biopsy at our institution (Children’s Hospital of Chongqing Medical University, Chongqing, P.R China). The decision to biopsy was at the discretion of the attending nephrologist. Core needle biopsy material was examined under the stereomicroscope and divided for light and electron microscopy studies. The sample for light microscopy was fixed in neutral buffered formalin was embedded in paraffin or optimal cutting temperature (OCT, 4583, SAKURA, America) compound by using standard procedures. Paraffin sections were stained with H&E, PAS, IHC and IF, respectively. Digital images were obtained with a light microscope (Olympus).

### Transmission electron microscopy (TEM)

Electron microscopic sample handling and detection were performed by the electron microscopic core lab of Chongqing Medical University. TEM images were analyzed using Image Pro plus 6.0. Four glomeruli were randomly selected and ten electron micrographs were taken in each glomerulus.

### Confocal and fluorescence microscopy

Kidney biopsies and 293 T cells fixed in neutral buffered formalin were embedded in paraffin or optimal cutting temperature compound by using standard procedures. Frozen and paraffin sections were stained with immunofluorescence, respectively. Immunofluorescent staining and images were obtained by a Nikon A1R Meta confocal microscope. Cover slips were observed.

The antibodies used were list below: anti-Cubilin-C-terminal antibody (1:500, ab191073, Abcam), Rat Cubilin(CUBN) polyclonal antibody (1:100, 31010, Bicell Scientific), anti-Synaptop-odin antibody (1:50, 21064-1-AP, Proteintech), anti-Wilms Tumor Protein antibody (1:50, ab89901, Abcam), anti-COL4A3 antibody (1:100, Kingmed, Guangzhou, China), anti-COL4A5 antibody (1:100, Kingmed, Guangzhou, China), anti-Amnionless antibody (1:10, sc-365384, Santa Cruz), anti-Megalin Antibody (1:30, CD7D5, Novus Biologicals), goat polyclonal secondary antibody to mouse Alexa fluor 488 (1:400, ab150113, Abcam), goat polyclonal secondary antibody to rabbit Alexa fluor 555 (1:400, ab150078, Abcam), rabbit monoclonal to HA tag (1:500, ab236632, Abcam), goat polyclonal secondary antibody to rabbit Alexa fluor 647 (1: 400, ab150079, Abcam), goat anti-mouse Alexa fluor 568 (1: 400, ab175473, Abcam), DAPI (1:1000, C1002, Beyotime).

### Cell culture

293 T cells were cultured in DMEM supplemented with 10% (v/v) FBS (Hyclone, 10100147) and 1% (v/v) penicillin/streptomycin (Beyotime, C0222) at 37 ℃and 5% CO^2^ in a humidified atmosphere and passaged every 2-3 days.

### Animals

Male BALB/c mice (20–22 g per mouse) and male C57BL6 mice (20–25 g per mouse) was kept under pathogen-free conditions at the Laboratory Animal Centre institution, Children’s Hospital of Chongqing Medical University (Chongqing, P.R China). After adaptive feeding for one week, BALB/c mice were injected by adriamycin (11 mg/kg, Meilunbio) through tail vein, and male c57 mice were administered intraperitoneally with Lipopolysaccharides (LPS, 12 mg/kg, Sigma-Aldrich). Control groups were received an equal volume of saline. BALB/c mice were anesthetized and sacrificed using isofluorane and euthanized by cervical dislocation at the fourth week after tail vein injection, while c57 mice were sacrificed in the same way at the 24th hour after intraperitoneal LPS injection. Kidney tissues were excised and fixed in 4% paraformaldehyde, embedded in paraffin. Paraffin-embedded sections were used to analyze the co-localization between Cubilin and Amn according to standard protocol. The experiment was approved by the Animal Ethics Committee of the Children’s Hospital of Chongqing Medical University (No. CHCMU-IACUC20220629011).

### Plasmid construction and transient transfection

The plasmids pLVX-IRES-ZsGreen1 and pCMV-HA-N were digested by EcoR I, respectively, and a 10,869 bp of human *CUBN* gene, a 1359 bp of human *AMN* gene, linearized pLVX-IRES-ZsGreen1 and pCMV-HA-N were purified. Then, *AMN* and pLVX-IRES-ZsGreen1, *CUBN* and pCMV-HA-N were linked utilizing In-Fusion Cloning (Vazyme, ClonExpress II One Step Cloning Kit, C112) to generate shuttle recombinant plasmids pLVX-IRES-ZsGreen1-AMN and pCMV-HA-N-CUBN. The shuttle plasmids were identified by Sanger sequencing analysis. Site-directed mutagenesis of *CUBN* were performed using Mut Express MultiS Fast Mutagenesis Kit V2(Vazyme, C215) and also identified by Sanger sequencing analysis.

The day prior to transfection, the cells were seeded into 24-well plates at 1 × 10^5^ cells/well. The cells were transfected using Lipofectamine3000 (ThermoFisher, USA) according to the manufacturer’s instructions with 500 ng of respective plasmid DNA per well. After 6–7 h, the medium was exchanged with fresh medium.

### Co-immunoprecipitation (Co-IP) assay and Western blot analysis

Protein extracts were prepared and incubated with anti-bodies against HA or IgG for 24 h on a rotating wheel. Then, Pierce Protein A/G Magnetic Beads (ThermoFisher, USA) were added and incubated for another 24 h. After the beads were boiled, the precipitated proteins were separated by SDS-PAGE and transferred to PVDF membranes for further analysis. For western blotting, cell samples were extracted and quantified then boiled at 95 ℃, 10 min. Protein sample was separated on a 6% sodium dodecyl sulfate polyacrylamide gel electrophoresis gel then transferred on a polyvinylidene fluoride (PVDF) membrane. Incubating primary antibodies overnight at 4 ℃, with specific primary antibodies against HA (1:1000, ab236632, Abcam), Cubilin (1:1000, ab191073, Abcam), anti-Amnionless antibody (1:500, sc-365384, Santa Cruz) and β-tubulin (AB0039, 1:2000, Abways) in Tris-Buffered Saline Tween-20 (TBST) containing 5% skim milk. After washed for 3 times with TBST, the membranes were incubated for 1 h at room temperature with a respective IgG-HRP labeled second antibody (1:10,000) in TBST containing 5% skim milk. Antigens were revealed using a chemiluminescence assay (Pierce ECL Western Blotting Substrate, 32,209, ThermoFisher, USA) and quantification of bands was achieved by densitometry using the Image J software.

### Proximity ligation assay

HEK 293 T cells were grown in 24-well plates containing coverslips (14 mm diameter) and cultured overnight. Then cells were treated with plasmid as described for transient transfection. Coverslips were washed with PBS twice and fixed in 4% paraformaldehyde for 15 min. Then coverslips were blocked with Duolink Blocking Solution for 60 min at 37 °C. The primary antibody HA and AMN, diluted in blocking solution, was added to the coverslips and incubated overnight at 4 °C. Then coverslips were washed with 1 × Wash Buffer A and subsequently incubated with Duolink^®^ PLA Probe (Duolink^®^ In Situ PLA^®^ Probe Anti-Rabbit PLUS, DUO92002, Duolink^®^ In Situ PLA^®^ Probe Anti-Mouse MINUS, DUO92004) for 60 min at 37 °C. The subsequent steps of ligation and amplification were performed according to the manufacturer's instructions (DUO92013, Sigma). Finally, coverslips were covered with Duolink In Situ Mounting Medium with DAPI (DUO82040, sigma). Images were obtained using Nikon A1 confocal microscope.

### Data availability

All data included in this study are available upon request by contact with the corresponding author.

## Results

### ES was used to identify novel compound heterozygous *CUBN* variants (NM_001081.3: c.4397G > A and c.6796C > T, c.6821 + 3A > G and c.5153_5154delCT)

The proband-1 was an eight-year-old Chinese boy with unexplained recurrent proteinuria since the age of two, and the proband-2 also had unknown proteinuria. Furthermore, most of the treatments for lowering proteinuria remained ineffective. Based on early onset and unclear proteinuria etiology, genetic kidney disease was clinically suspected. Therefore, ES was performed to screen for strong candidate genes underlying susceptibility loci for the recurrent proteinuria and the schematic diagram of the workflow for screening pathogenic mutations was shown in Additional file 1: Fig. S1. ES performed in proband 1 showed variants of unknown significance in the following genes: *ANLN* (associated with FSGS), NM_018685, hemizygous c.2748 + 6 T > C (splicing); *CLCNKB* (associated with Bartter Syndrome Type 3 and Type 4B clinically featured by hypokalaemic metabolic alkalosis) hemizygous c.118delA (NM_001165945) and c.782-10_782-8delCCT (splicing, NM_000085) [18–20]. Given these variants exhibiting symptoms that does not fall within the above clinical phenotypes of proband 1, we excluded the possibility. Finally, it was found that the variants (NM_001081, c.4397G > A (p.C1466Y) and c.6796C > T (p.R2266X)) in *CUBN* may be responsible for unknown proteinuria. Meanwhile, analysis of ES on proband 2 showed that only 2 suspicious variants (NM_001081, c.5153_5154delCT (p.S1718X) and c.6821 + 3A > G (splicing)) in *CUBN* were highly correlated with the above clinical phenotypes. To this end, we identified novel compound heterozygous *CUBN* variants with biallelic, likely pathogenic variants, segregating with proteinuria (Table 1 and Additional file 2: Table S1). All the variants were not listed in the clinvar database, and the minor allele frequency (MAF) was less than 0.01 (Table 1).

**Table 1:** The information about SNPs of *CUBN* gene from the index patient including minor allele frequency (MAF) and inheritance from parents

Chromosome position	MAF	Base	Gene	Function	Type of mutation	ClinVar assessment	Inheritance
		(amino acid)					
chr10:16961987	0.000077	c.6796C > T	*CUBN*	Nonsense	Germline	Not listed	Father
		(p.R2266X)					
chr10:17026232	0.000077	c.4397G > A	*CUBN*	Missense	Germline	Not listed	Mother
		(p.C1466Y)					
chr10:169	–	c.5153_5154delCT	*CUBN*	Frame shift	Germline	Not listed	Father
90531–16990533		(p.S1718X)					
chr10:16961959	0.0013	c.6821 + 3A > G	*CUBN*	Splice variant	Germline	Not listed	Mother
		(splicing)					

**Fig. 1:**
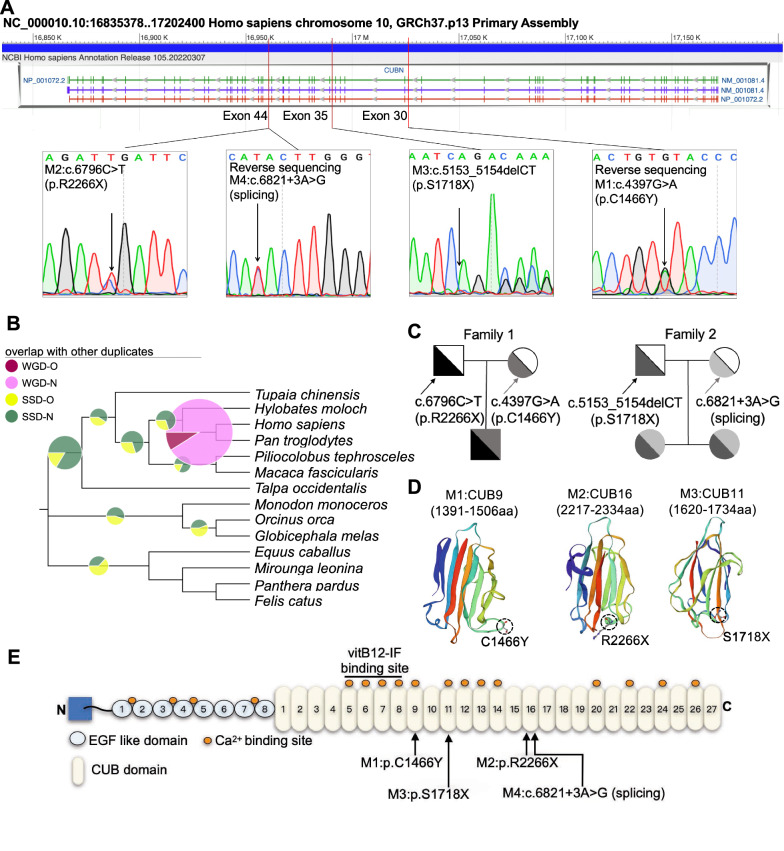
Identification of novel compound heterozygous variants at *CUBN* using ES in two families. **A** Schematic to scale overview of the genomic *CUBN* structure and the variants (c.6796C > T and c.4397G > A, c.6821 + 3A > G and c.5153_5154delCT) were mapped. And then the variants were further confirmed by Sanger sequencing in the two probands’ families. **B** Phylogenetic analysis of *CUBN*. The proportion of replicate trees in which the associated taxa clustered together in the bootstrap test were shown next to the branches. **C** Pedigree of the probands’ family with the novel compound heterozygous variants in *CUBN* gene. **D** Crystal structures of CUB domain 9, 11 and 16 of Cubilin protein oligomers in the oligomerized conformation. The mutated residues (p.C1466Y, p.S1718X and p.R2266X) were marked with Dotted circles. **E** Schematic of the Cubilin protein domains. The novel variants were marked with black arrows

Subsequently, the variations were verified by Sanger sequencing (Fig. 1A). Phylogenetic *CUBN* analysis in different mammalian species showed the gene originated from a common ancestry root, and evolutionary diverged into three major clades suggesting interspecies *CUBN* conservation was high (Fig. 1B). In light of the genetic predisposition, Sanger sequencing of parents of the probands, who exhibited normal phenotypes, confirmed the variants were inherited from them (Fig. 1C). Interestingly, the sister of proband-2 carried the same variants as the proband and presented with proteinuria as well. Three-dimensional structural models showed that both variants potentially affected folding and function of CUB9, CUB11, CUB16 domains (Fig. 1D). Furthermore, Cubilin functional domain analysis identified the heterozygous C1466Y and S1718X variants were near the back of vitB12-binding domain, whereas the p.R2266X variant and c.6821 + 3A > G variants were in the C-terminal CUB16 region, four of which were shown to have proteinuria without vitB12 malabsorption (Fig. 1E and Table 2).

**Table 2:** Related serological biochemical parameters detection as albumin, blood lipid profiles and vitB12

Characteristic	Reference range	Result	
		Proband 1	Proband 2
Albumin	38–55 g/L	45	41.4
Triglyceride	0.3–1.8 mmol/L	1.45	0.42
Total cholesterol	2.7–5.5 mmol/L	3.99	3.27
Low density lipoprotein cholesterol	0–3.1 mmol/L	2.16	1.74
High density lipoprotein cholesterol	0.91–2.04 mmol/L	1.63	1.32
Vitamin B12	211–911 pg/ml	521	372

Thus, these results suggested that there may be an association between the novel variants locating after vitB12-binding domain of *CUBN* and chronic proteinuria.

### Different mutation types after vitB12-binding domain in *CUBN* had similar functional effects on Cubilin

**Fig. 2:**
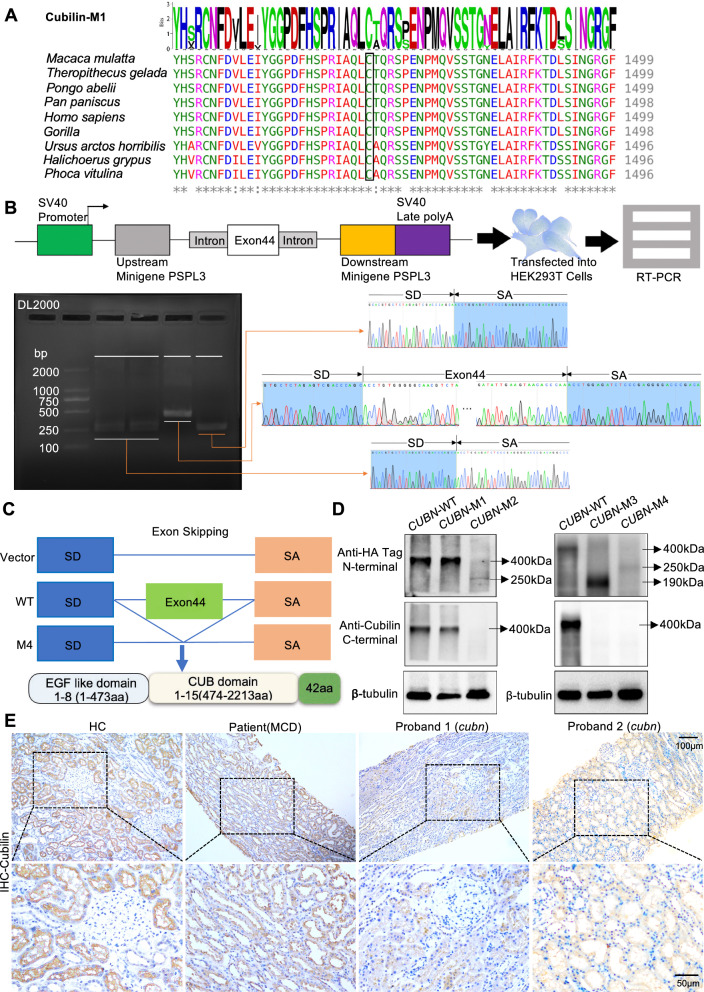
The novel variants with different variants types locating after vitB12-binding domain are related to partial dysfunction of Cubilin. **A** ClustalW multiple sequence alignment of Cubilin protein in several species. The novel missense variant (p.C1466Y) identified in one proband was located at a highly conserved position in Cubilin protein, as highlighted in black box. The asterisk signs below the sequence alignment indicate evolutionary conserved residue, the colon signs indicate highly conserved residue and the period signs represent less conserved residue. **B** *CUBN* minigene splice assay. The pSPL3 reporter minigene construct used in this functional assay and subcloning of the genomic *CUBN* fragment from wild- type and mutant alleles. RT-PCR analysis of transcripts derived from the indicated reporter assay in HEK293T cells and sequence analysis of the electrophoresis gel recovery product. **C** Sequencing of the above bands revealed that the splicing variant (c.6821 + 3A > G) resulted in exon 44 skipping and early termination of the amino acids. **D** Western blot analysis for Cubilin expression of the four variants. **E** Immunohistochemistry (IHC) staining showed the expression of Cubilin in *cubn* probands’ kidney biopsies exhibited markedly decline when compared to MCD and HC. M1: *CUBN* (c.4397G > A), M2: *CUBN* (c.6796C > T), M3: c.5153_5154delCT and M4: c.6821 + 3A > G

To assess effects of the variants on Cubilin dysfunction respectively, we first performed an amino acid conservation analysis on the missense variants site (p.C1466Y). Using ClustalW multiple sequence alignments, C1466Y was localized to an evolutionary, highly conserved region in the Cubilin CUB9 domain (Fig. 2A). As the minigene technology was confirmed as a reliable tool to functionally assay potential splicing variants, we checked the c.6821 + 3A > G variants to dissect alternative splicing effects using pSPL3 vector in HEK-293 cells. The splice variant showed skipping of exon 44 to generate premature termination codon (Fig. 2B and C).

To further verify variants associations with protein expression and functions, we constructed recombinant plasmids expressing Cubilin (wild type), Cubilin (p.C1466Y) and the truncated Cubilin (p.S1718X, p.R2266X and p.I2654X), and transiently transfected these into Human Embryonic Kidney 293 T cells. We then performed western blotting to assess Cubilin expression. The mutant *CUBN* (c.4397G > A) M1 plasmid expressed normal Cubilin, similar to Cubilin (wild type). However, the mutant *CUBN* (c.6796C > T) M2 disrupted the C-terminus, and truncated Cubilin expression (Fig. 2D). It was the same with the mutant *CUBN* (c.5153_5154delCT, p.S1718X) M3 and *CUBN* (c.6821 + 3A > G, p.I2654X) M4 (Fig. 2D).

Furthermore, renal biopsies from a patient (similar age to the proband) with clinically diagnosed non-hereditary MCD, and adjacent healthy kidney tissue from healthy kidney donor who received living-related donor nephrectomy as healthy control (HC) were used to assess in vivo Cubilin expression by immunohistochemistry (IHC). We observed a marked decline in Cubilin expression in the proband’s kidney biopsy when compared with HC and MCD samples (Fig. 2E). Therefore, different variants after vitB12-binding domain in *CUBN* all appeared to be associated with dysfunctional Cubilin.

### The variants of Cubilin exhibited normal GFB

**Fig. 3:**
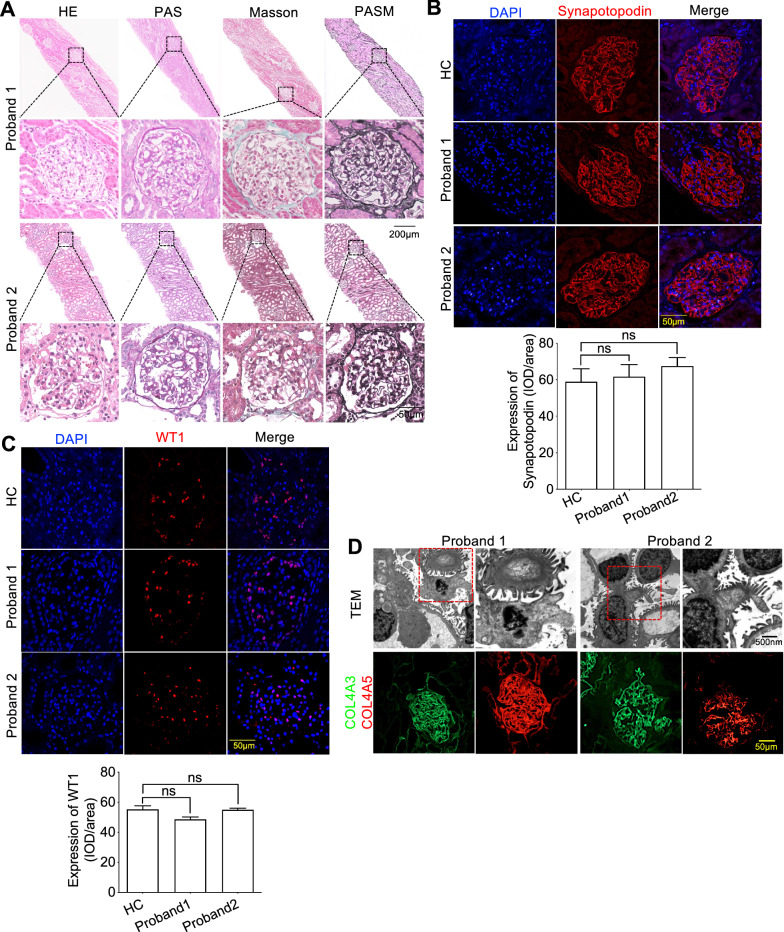
Compound heterozygous variants locating after vitB12-binding domain of *CUBN* exhibited normal GFB. **A** hematoxylin–eosin (HE), periodic acid–Schiff (PAS), periodic acid-silver metheramine (PASM) and Masson of kidney biopsy of the two probands showed there was no obvious proliferation of glomerular mesangial cells, inflammatory cell infiltration, fibrosis, glomerular sclerosis or segmental sclerosis. **B** and **C** Immunofluorescence staining showed the expression of podocyte membrane marker Synapotopodin and nuclear marker WT1 was normal as compared to HC. **D** Representative photomicrographs by transmission electron microscopy (TEM) analyses showed there were no thickening of GBM or widening and fusion of podocytes and immunofluorescence staining with COL4A3 and COL4A5, the important components of GBM, indicated that there was no exact immune complex deposition. Each group was tested in triplicate, and the data are presented as the mean ± S.D. ns, no significant

To investigate if proteinuria was caused by glomerular damage, percutaneous renal biopsies from the two probands were analyzed by hematoxylin & eosin (H&E), periodic acid–Schiff (PAS), periodic acid-silver metheramine (PASM), and Masson staining. Thankfully, we observed no glomerular and segmental sclerosis in both probands (Fig. 3A). Next, we performed immunofluorescence (IF) using Synaptopodin (Synpo) (Fig. 3B) (a podocyte marker and actin-associated protein in podocyte foot processes) and a Wilms' tumor-1 (WT-1) podocyte nuclear marker (Fig. 3C). IF with these markers indicated no damage or podocyte loss. Furthermore, transmission electron microscopy (TEM) identified multifocal gaps in the GBM, with no electron-dense deposits (Fig. 3D). Also, glomeruli did not present with podocyte foot broadening or effacement (Fig. 3D). Further IF staining was performed to confirm GBM scaffold structural integrity; COL4A3 and COL4A5 are glomerular filtration function markers, and IF staining with these markers showed no structural changes in the GBM (Fig. 3D).

Thus, the variants after vitB12-binding domain of *CUBN* variants exhibited normal renal function as evidenced by normal glomerular structures.

### The variants of Cubilin only associated with aberrant renal tubular protein reabsorption

As indicated, *CUBN* variants caused no pathological structural changes in the glomerulus. To address the cause of proteinuria, we tracked renal function and measured serum autoantibodies and urine protein excretion over 24 h (Tables 3 and Additional file 2: Table S2). Of the autoantibodies associated with clinically relevant renal autoimmune disease, membranous nephropathy (MN), lupus nephritis (LN), and anti-neutrophil cytoplasmic autoantibody (ANCA)-associated vasculitis (AAV) [21] have been detected. These studies suggested neither of the probands had autoimmune dysfunction. We also observed that the urinary albumin-to-creatinine ratio (UACR) was significantly high (Table 3). However, in spite of these high levels, no obvious abnormalities in total urinary protein excretion were identified (Table 3). Of note, α1-microglobulin and transferrin, which are urine biomarkers of kidney tubule injury and dysfunction [22, 23], were significantly increased, while β2-microglobulin was normal (Table 3).

**Table 3:** Detection of biomarkers from proband urine

Characteristic	Reference	Result	
	Range	Proband 1	Proband 2
Urinary albumin	< 150 mg/L	297.85 ± 23.23	527.98 ± 384.37
Urinary creatinine	–	8.91 ± 1.57	26.514
Total 24-h urinary protein	< 230 mg/24 h	414 ± 166.88 ↑	485 ± 190.92 ↑
Urinary albumin/urinary creatinine	0–30 mg/g	300.86 ± 42.18 ↑	363 ↑
Urinary α1-microglobulin	< 12 mg/L	27.08 ± 10.48 ↑	16.65 ± 8.25 ↑
Urinary β2-microglobulin	< 0.3 mg/L	0.18 ± 0.08	0.1 ± 0.25
Urinary transferrin	< 2 mg/L	21.60 ± 2.16 ↑	39.26 ± 31.52 ↑

The black arrows represent the increase of corresponding detection indicators

**Fig. 4:**
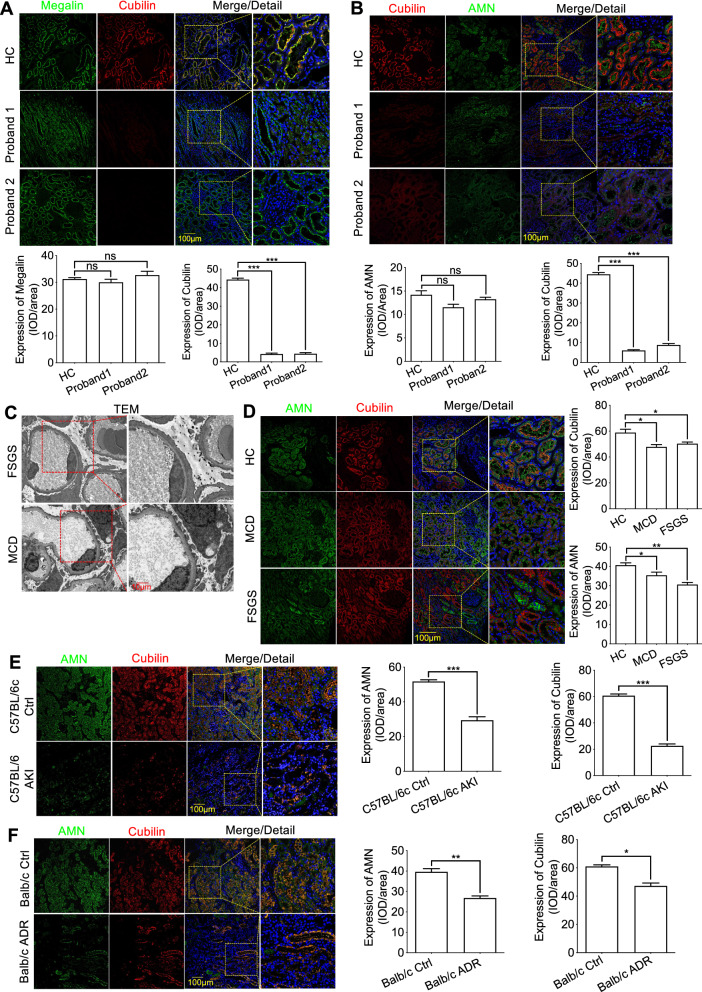
Dysfunction of Cubilin induced by the variants locating after vitB12-binding domain of *CUBN* was accompanied by abnormal localization of AMN. **A** The protein expression of Cubilin and Megalin in kidney tissues was detected by immunofluorescence assay and showed the expression and localization of Megalin did not accompany the decrease of Cubilin expression. **B** Immunofluorescence staining showed the decrease expression of Cubilin caused by the *CUBN* variant was accompanied by aberrant cytoplasmic localization of AMN in renal tubule membrane. **C** and **D** Representative photomicrographs by TEM analyses confirmed the GFB damage and immunofluorescence staining were to detect the colocalization and expression between Cubilin and AMN in kidney biopsies from MCD and FSGS. **E** and **F** The reduced expression of renal Cubilin was not accompanied by abnormal localization of AMN in the mice model of LPS-induced acute kidney injury and Adriamycin-induced nephropathy. Each group was tested in triplicate, and the data are given data are presented as the mean ± S.D. ns, no significant; *P < 0.05; **P < 0.01; ***P < 0.001

According to the literature, Cubilin is an important receptor, possibly mediating albumin, transferrin and α1-microglobulin endocytosis into renal tubules [24–26]. When we further considered the probands’ clinical observations, we hypothesized the Cubilin variants may have been involved in renal tubule dysfunction and aberrant renal tubule protein reabsorption, possibly accounting for the chronic isolated proteinuria. To establish a relationship between genetic variants and chronic proteinuria, IF was used to assess Cubilin, Megalin, and AMN co-expression. Interestingly, the variants did not induce aberrant Megalin expression or localization when compared with the HC (Fig. 4A). However, decreased Cubilin expression (caused by the variants) was accompanied by aberrant cytoplasmic localization of AMN in renal tubule membranes (Fig. 4B).

From these results, we hypothesized if different levels of GFB dysfunction may have affected Cubilin and AMN colocalization. To this end, we first explored the colocalization of Cubilin and AMN in kidney biopsies in MCD and FSGS that were identified as non-hereditary nephropathy of GFB damage with similar age. Further TEM was used to investigate podocyte damage, with varying degrees of segmental podocyte foot process broadening, fusion, and effacement in MCD and FSGS, being consistent with the characteristics of the diseases (Fig. 4C). Surprisingly, neither Cubilin nor AMN showed aberrant localization (Fig. 4D). Additionally, in vivo experiments further confirmed that the reduced expression of renal Cubilin did not cause abnormal cytoplasmic localization of AMN in the mice model of LPS-induced acute kidney injury and Adriamycin-induced nephropathy with GFB impairment (Fig. 4E and F). These observations suggested that GFB dysfunction may not have been the cause of aberrant cytoplasmic localization of AMN.

### Cubilin dysfunction induced by the variants was accompanied by aberrant cytoplasmic localization of AMN

**Fig. 5:**
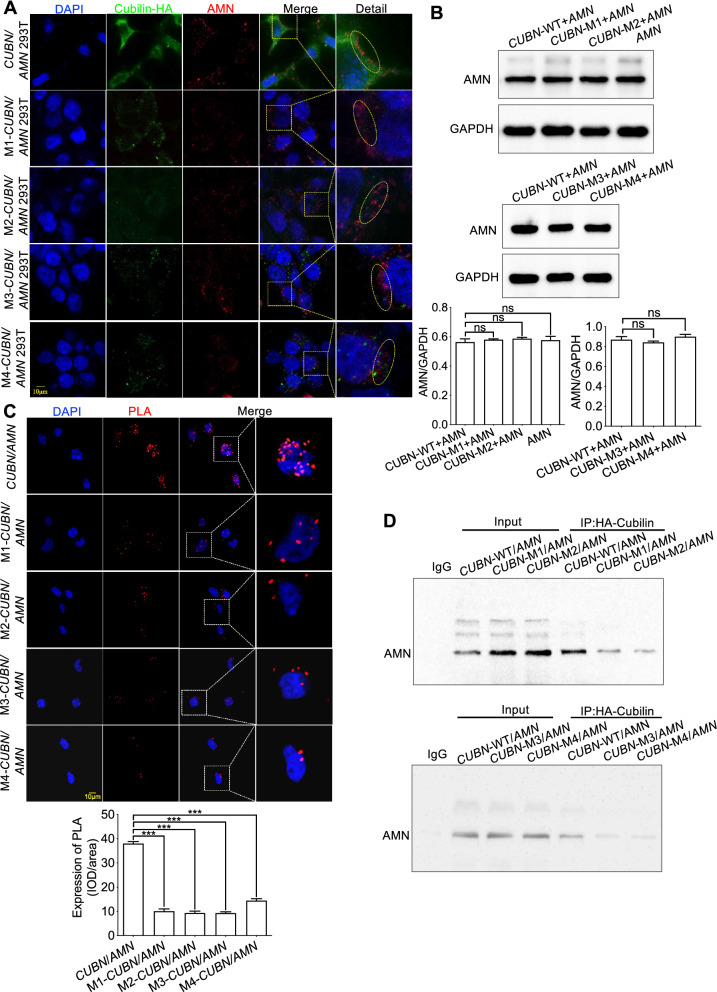
The variants locating after vitB12-binding domain of Cubilin affected the localization of AMN. **A** Human 293 T cells co-transfected with eukaryotic transient-expression plasmids (*AMN* with *CUBN*, M1-*CUBN*, M2-*CUBN*, M3-*CUBN* and M4-*CUBN* respectively) indicated the four variants affected Cubilin expression accompanied by abnormal localization of AMN. **B** Western blot analysis for AMN expression of the co-transfected recombinant plasmids. **C** and **D** Co-IP and Duolink proximity ligation assay were used to analysis the protein interaction between AMN and the variants of Cubilin. Each group was tested in triplicate, and the given data are presented as the mean ± S.D. ns, no significant; ***P < 0.001

To elucidate the colocalization relationship between Cubilin and AMN, HEK293T cells were co-transfected with eukaryotic transient-expression plasmids (*AMN* with *CUBN*, M1-*CUBN*, M2-*CUBN*, M3-*CUBN* and M4-*CUBN*). Cubilin was localized to the cell membrane, and AMN co-localized with Cubilin under normal physiological conditions (Fig. 5A). However, the four Cubilin variants all caused aberrant AMN localization, and retained it to the cytoplasm (Fig. 5A). Subsequently, western blot was employed to further investigate the expression of AMN and the results indicated that the variants of Cubilin may only cause abnormal localization of AMN by affecting the protein interaction with AMN, but does not affect its expression (Fig. 5B). To this end, we further tested the interaction between the variants of Cubilin and AMN using co-immunoprecipitation (co-IP) and Duolink proximity ligation assay (Fig. 5C and D). The results further demonstrated that the variants of Cubilin reduced the ability to interact with AMN.

Altogether, the domain locating after vitB12-binding of Cubilin was essential for AMN localization and could provide a scaffold to maintain AMN interactions in membrane, whereas variants after vitB12-binding of *CUBN* only promoted aberrant AMN localization without affecting its expression.

## Discussion

CKD is an important global public health problem, constituting a major health burden [27, 28]. Of note, genetic factors are important in CKD etiology, and are especially concerning in children with CKD [29, 30]. ES is a proven diagnostic method for CKD; it works well with genetic and phenotypic heterogeneity in hereditary nephropathies, and exemplifies how genetic testing can resolve clinical diagnostic challenges [8]. Proteinuria is a common clinical manifestations of kidney injury and is an independent risk factor for CKD progression [31, 32]. However, it is unclear if all proteinuria forms are caused by genetic factors and are damaging to the patient, and the knowledge gaps remain.

In this study, we identified two probands with isolated proteinuria and associated with different variants locating after the vitB12-binding domain of *CUBN*. The variants only led to declined Cubilin expression accompanied by aberrant AMN localization in renal tubules, of which localization depended on Cubilin function to maintain correct renal tubule protein reabsorption. However, we found variants locating after vitB12-binding of *CUBN* caused Cubilin to lose its scaffolding capabilities, resulting in aberrant AMN localization in cytoplasm. When combined with the clinical findings, we hypothesized that the different variants locating after the vitB12-binding domain of *CUBN* accounted for chronic isolated proteinuria in this patient, without GFB dysfunction, vitB12 deficiencies or abnormal blood levels of HDL and albumin.

We partly identified the contribution of domain polymorphism locating after the vitB12-binding domain of Cubilin to AMN localization. A previous study reported that AMN depended on Cubilin for correct localization [33] and this Cubilin/AMN interdependency helped maintain renal tubule protein reabsorption [34–36]. In addition, we showed that AMN is a chaperone for Cubilin; the domain locating after vitB12-binding domain of Cubilin attaches great importance to provide a membrane scaffold for AMN for maintaining renal tubule protein reabsorption. Therefore, *CUBN* polymorphism locating after the vitB12-binding domain may be related to an increased incidence and risk of proteinuria associated with renal tubule dysfunction. However, the in-depth characterization of a molecular chaperone mechanism requires more research. One possible molecular explanation for AMN failure to localize to the membrane could be that scaffold destruction of the domain locating after the vitB12-binding domain impairs AMN structural modification leading to it retaining in the cytoplasm. Further studies should elucidate these specific regulatory mechanisms.

An unexpected finding was that the endocytic receptor, Megalin, displayed normal localization and expression with decreased Cubilin expression. However, Cubilin is a transferrin receptor and mediates endocytosis in a Megalin-dependent manner [24]. Functionally, it was reported that Megalin contributed to increasing uptake of intrinsic factor-vitB12 complex, mediated by Cubilin-AMN complexes, of which the main role in albumin reabsorption is to drive the internalization of the complexes [25]. Unlike the more N-terminal IGS variants, the Cubilin variants in our study led to modifications or truncations after the vitB12-binding domain that may partly explain that the absorption of vitB12 is normal.

## Conclusions

In conclusion, we proposed the different variants locating after the vitB12-binding domain of *CUBN* caused non-detrimental chronic isolated proteinuria as albuminuria, increased in urine transferrin and α1-microglobulin levels, with normal levels of urine β1-microglobulin levels, intact GFB, blood levels of HDL and albumin and vitB12 absorption, that may not require any proteinuria-lowering treatment or renal biopsy. Importantly, our data provided key genetic insights into CKD pathogenesis and identify potential therapeutic approaches for chronic isolated proteinuria. Also, the insights raise the possibility to provide valuable information for genetic counseling and prenatal diagnostics.

## Supplementary Information

**Additional file 1: Figure S1.** Schematic diagram of the workflow for screening pathogenic mutations.

**Additional file 2: Table S1.** Variants in gene *CUBN* and their predicted properties of mutant protein. **Table S2.** Serological assays including immunity indicators.

## Data Availability

All data and materials included in this study are available upon request by contacting with the corresponding author.

## Abbreviations

CKD: Chronic kidney disease
AMN: Amnionless
ESRD: End-stage renal disease
GBM: Glomerular basement membrane
ES: Exome sequencing
MCD: Minimal-change disease
FSGS: Focal segmental glomerulosclerosis
HC: Health control
IHC: Immunohistochemistry
IF: Immunofluorescence
UACR: Urinary albumin-to-creatinine ratio
Synpo: Synaptopodin
WT1: Wilms' tumor-1
