# Supplementary figures and images for: Novel pathogenic variants in CUBN uncouple proteinuria from renal function
Source: J Transl Med. 2022 Oct 20;20:480. doi: 10.1186/s12967-022-03706-y (PMC9583559; doi:10.1186/s12967-022-03706-y)

Supplemental Figure 1

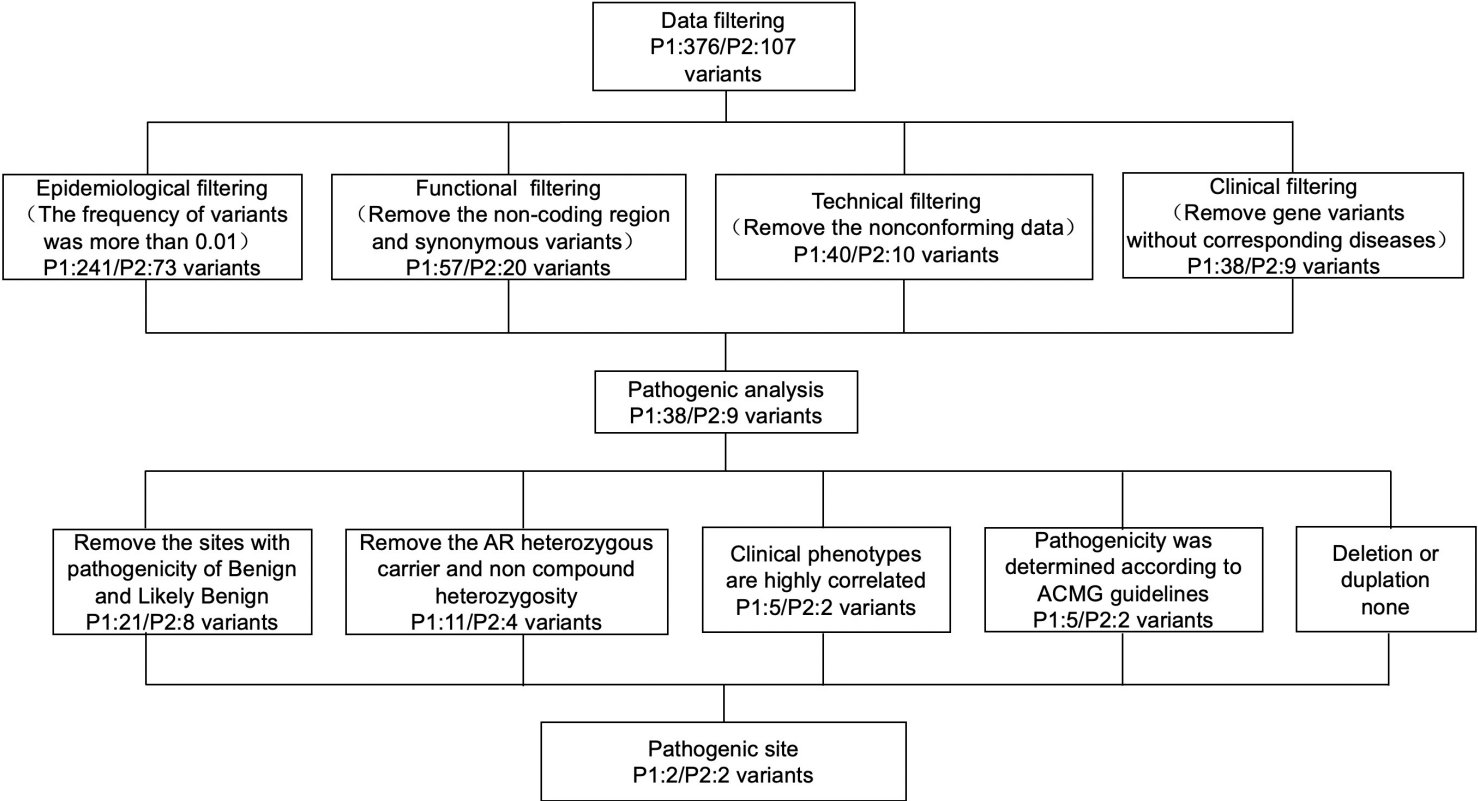

Supplement: Supplementary file 1 — Additional file 1: Figure S1. Schematic diagram of the workflow for screening pathogenic mutations. [file 12967_2022_3706_MOESM1_ESM.pdf]
