# Supplementary material for: Novel pathogenic variants in CUBN uncouple proteinuria from renal function
Source: J Transl Med. 2022 Oct 20;20:480. doi: 10.1186/s12967-022-03706-y (PMC9583559; doi:10.1186/s12967-022-03706-y)
Supplement: Supplementary file 2 — Additional file 2: Table S1. Variants in gene CUBN and their predicted properties of mutant protein. Table S2. Serological assays including immunity indicators. [file 12967_2022_3706_MOESM2_ESM.pptx]

## Slide 1
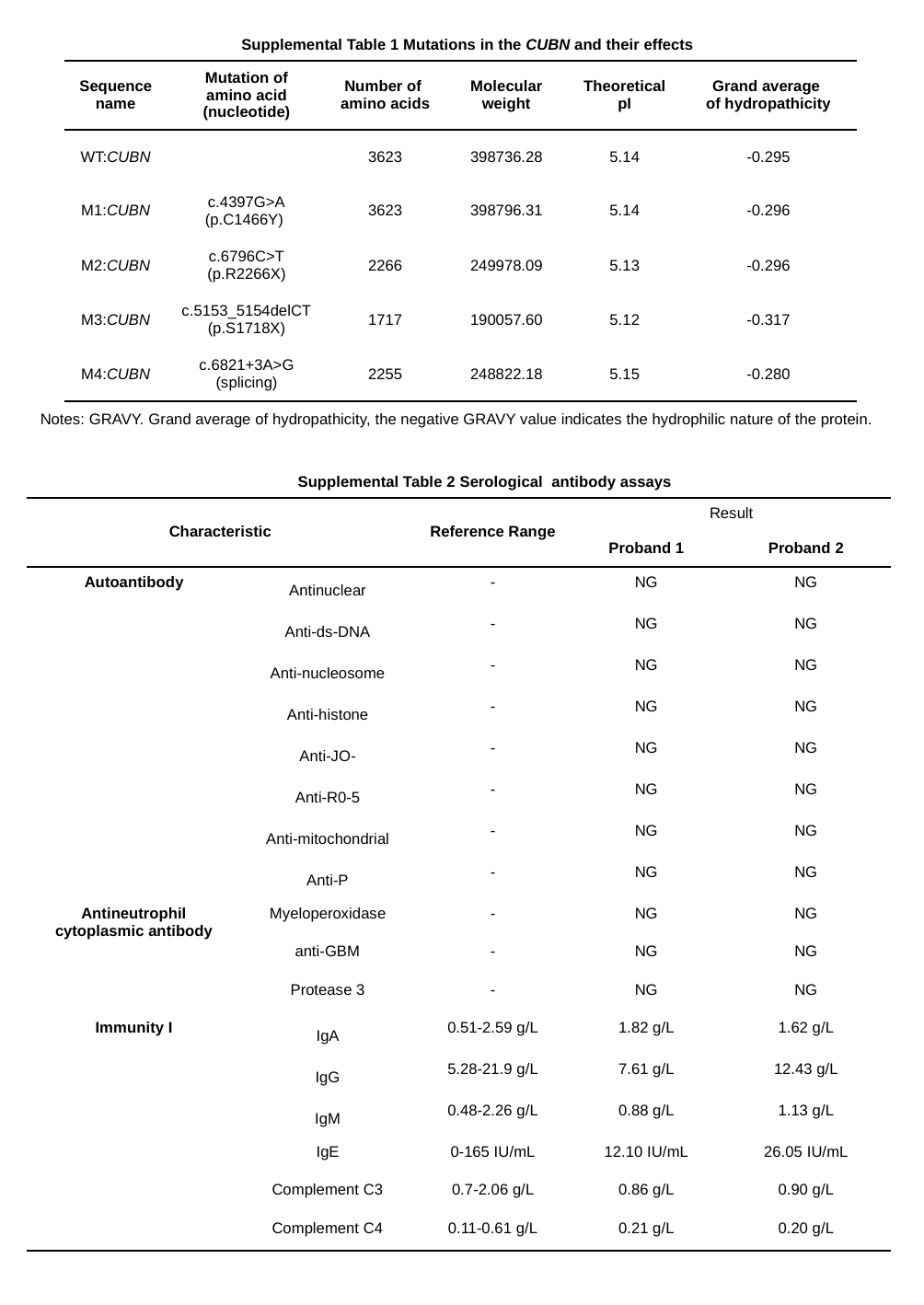

Supplemental Table 1 Mutations in the CUBN and their effects
| Sequence name | Mutation of amino acid (nucleotide) | Number of amino acids | Molecular weight | Theoretical pI | Grand average of hydropathicity |
| --- | --- | --- | --- | --- | --- |
| WT:CUBN | | 3623 | 398736.28 | 5.14 | -0.295 |
| M1:CUBN | c.4397G>A (p.C1466Y) | 3623 | 398796.31 | 5.14 | -0.296 |
| M2:CUBN | c.6796C>T (p.R2266X) | 2266 | 249978.09 | 5.13 | -0.296 |
| M3:CUBN | c.5153\_5154delCT (p.S1718X) | 1717 | 190057.60 | 5.12 | -0.317 |
| M4:CUBN | c.6821+3A>G (splicing) | 2255 | 248822.18 | 5.15 | -0.280 |
Notes: GRAVY. Grand average of hydropathicity, the negative GRAVY value indicates the hydrophilic nature of the protein.
Supplemental Table 2 Serological antibody assays
| Characteristic | | Reference Range | Result | |
| --- | --- | --- | --- | --- |
| | | | Proband 1 | Proband 2 |
| Autoantibody | Antinuclear | - | NG | NG |
| | Anti-ds-DNA | - | NG | NG |
| | Anti-nucleosome | - | NG | NG |
| | Anti-histone | - | NG | NG |
| | Anti-JO- | - | NG | NG |
| | Anti-R0-5 | - | NG | NG |
| | Anti-mitochondrial | - | NG | NG |
| | Anti-P | - | NG | NG |
| Antineutrophil cytoplasmic antibody | Myeloperoxidase | - | NG | NG |
| | anti-GBM | - | NG | NG |
| | Protease 3 | - | NG | NG |
| Immunity I | IgA | 0.51-2.59 g/L | 1.82 g/L | 1.62 g/L |
| | IgG | 5.28-21.9 g/L | 7.61 g/L | 12.43 g/L |
| | IgM | 0.48-2.26 g/L | 0.88 g/L | 1.13 g/L |
| | IgE | 0-165 IU/mL | 12.10 IU/mL | 26.05 IU/mL |
| | Complement C3 | 0.7-2.06 g/L | 0.86 g/L | 0.90 g/L |
| | Complement C4 | 0.11-0.61 g/L | 0.21 g/L | 0.20 g/L |
